# The Effect of 50 000 IU Vitamin A with BCG Vaccine at Birth on Growth in the First Year of Life

**DOI:** 10.1155/2011/570170

**Published:** 2011-09-08

**Authors:** Ane Bærent Fisker, Christine Stabell Benn, Birgitte Rode Diness, Cesario Martins, Amabelia Rodrigues, Peter Aaby, Bo Martin Bibby

**Affiliations:** ^1^Bandim Health Project, Indepth Network, Apartado 861, 1004 Bissau, Guinea-Bissau; ^2^Bandim Health Project, Statens Serum Institut, Ørestads Boulevard 5, 2300 Copenhagen S, Denmark; ^3^Department of Biostatistics, Institute of Public Health, University of Århus, Bartholins Alle 2, 8000 Århus C, Denmark

## Abstract

Vitamin A supplements may interact with diphtheria-tetanus-pertussis (DTP) vaccine causing increased female mortality. In a randomised trial of neonatal vitamin A supplementation (VAS), we examined growth during the first year of life in 808 children, pursuing the hypothesis that a negative interaction between VAS and DTP in girls would be reflected in growth. Length and weight were measured at 6 weekly visits and WHO-growth-reference z-scores derived. Neonatal VAS had no effect on anthropometric measures at 12 months, but may interact sex differentially with routine vaccines. While BCG was the most recent vaccine, neonatal VAS benefitted growth (difference in weight-for-length z-score (dWFL: 0.31(95% CI: 0.03–0.59)). While DTP was the most recent vaccine, VAS tended to affect growth adversely in girls (dWFL = −0.21 (−0.48–0.06)). After measles vaccine (MV) there was no overall effect of neonatal VAS. The VAS effect differed significantly between the BCG and DTP windows (*P* = 0.03), and the difference was borderline significant between the DTP and MV windows for girls (*P* = 0.09).

## 1. Introduction

Vitamin A deficiency (VAD) has been associated with impaired growth in observational studies [1, 2], but randomised trials of vitamin A supplementation (VAS) have often failed to show an impact on growth [3] in spite of improved vitamin A status in deficient populations [4, 5]. The only study examining the effect of neonatal VAS on growth reported a positive effect on height at 3 years of age in Indonesia [6]. Within a randomised placebo-controlled study of the effect on mortality of providing neonates with 50 000 IU of vitamin A with Bacillus Calmette-Guerin vaccine (BCG) [7] we examined the effect of neonatal VAS on growth during the first year of life in both sexes. 

The present trial as well as a similar trial of neonatal VAS from Zimbabwe [8] did not find the expected beneficial effect of VAS on mortality observed in Indonesia [9] and India [10] and later in Bangladesh [11]. We have previously hypothesised that VAS and diphtheria-tetanus-pertussis (DTP) vaccines may interact negatively [12, 13], due to amplification of the negative nonspecific effects of DTP vaccines. 

The nonspecific effects are the effects of vaccines that cannot be ascribed to the protection against the targeted disease(s). For example, observational studies [14, 15] and randomised trials [16, 17] of measles vaccine (MV) indicate reductions in overall mortality far bigger than the reduction due to prevention of measles infection. Similarly for BCG, reductions in mortality that cannot be ascribed to prevention of tuberculosis have been seen in randomised [18, 19] and observational studies [20]. The opposite effect is seen after DTP vaccinations; in areas with herd immunity to pertussis, DTP has been associated with increased female mortality reflected in increased mortality in DTP vaccinated compared to unvaccinated children [15] and increased female-male mortality rate ratio after DTP vaccination [21, 22]. In the present cohort we found that girls had 2-fold higher mortality after reception of DTP vaccine if they had received VAS rather than placebo at birth [23]. In the present study, we therefore examined whether VAS administered with BCG at birth affected growth during the first year of life and furthermore whether neonatal VAS interacted with subsequent routine vaccines. All analyses were stratified by sex since neonatal VAS may have sex-differential effects [7, 9, 24].

## 2. Subjects and Methods

### 2.1. Study Population

Bandim Health Project runs a Health and Demographic Surveillance System (HDSS) in six suburban districts of the capital of Guinea-Bissau, Bissau. The present study was carried out within a neonatal VAS trial which has been described in detail elsewhere [7]. Briefly, mothers who gave birth to infants of at least 2500 g were invited to participate in the trial when the child was due to be BCG vaccinated at the maternity wards or health centres. A trained assistant explained the study. Provided maternal consent, children were randomised to vitamin A or placebo. VAS was administered orally as 0.5 mL of oil containing 50 000 IU vitamin A as retinyl palmitate and 10 IU vitamin E and placebo was 0.5 mL of oil containing only 10 IU vitamin E. The code was broken when all enrolled children had reached 12 months of age.

### 2.2. Anthropometrics

The effect on growth was studied in a subcohort comprising all children aged 0 to 6 weeks of age when enrolled between July 1, 2004 and November 28, 2004, when the main trial ended. Hence, all children had been enrolled in the rainy season from June to November. We aimed to include 800 children. Children were visited every 6 weeks. Anthropometric measurements were initiated October 20, 2004. The oldest of these children were therefore measured for the first time at 3 months, corresponding to having missed one visit by the anthropometry team. 

Measurements were made by two trained field assistants who visited the home of the child. The length of the child was measured supine using a wooden measuring board. The weight of the undressed child was measured to the nearest 20 g using an electronic scale (SECA Model 835). Children, who were temporarily absent when the house was visited, were visited later the same or the following day, whereas children travelling were only visited at the following round. Children who moved within the study area were localized using the HDSS and visited at the new address.

### 2.3. Vaccine Information

**Figure 1:**
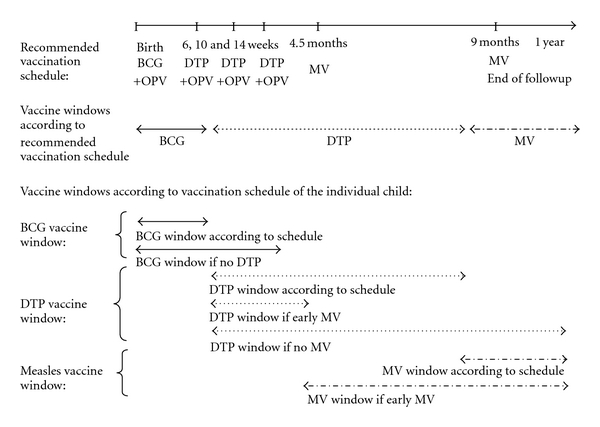
Vaccinations schedule and vaccine windows for infants in Guinea-Bissau.

The recommended immunisation schedule in Guinea-Bissau for normal-birth-weight infants during the conduct of the study was BCG and oral polio vaccine (OPV) at birth, 3 doses of DTP and OPV at 6, 10, and 14 weeks, and MV at 9 months of age (Figure 1). The actual timing of the vaccines varied depending on when the mother took the child to the health centre for vaccination. Due to periods with lack of OPV, some children received only BCG at birth. This was registered on the inclusion form. The anthropometry team collected information on vaccination status of the child when conducting the measurements. Furthermore, vaccination status was assessed through the HDSS at the home visits every 3 months, and all vaccines administered at the health centres in the study area are registered daily. The vaccine dates collected by the anthropometry team have been validated against these routine data.

**Early Measles Vaccine Trial**

Some of the children in the present VAS at birth trial were later enrolled in an early measles vaccine trial. Within that trial children, who had received all three DTP vaccines, were randomised to early MV at 4.5 months of age or no early MV. All children received MV at 9 months of age as recommended [17].

### 2.4. Analytical Strategy and Statistics

We used two complementary approaches to assess growth patterns in the first year of life. All analyses were conducted by sex as neonatal VAS may have sex-differential effects [24, 25]. For the both approaches we compared the individual measurements to the 2006 WHO growth reference [26]. Z-scores, that is, the number of standard deviations that a measurement differs from the reference curve for length for age, weight for age and weight for length were derived and children were classified as stunted (length-for-age z-score <−2) and underweight (weight-for-age z-score <−2) at all measured time points. Our first approach was to study whether neonatal VAS affected weight for age and length for age at 6 months (when the children would be eligible to receive VAS in campaigns; range 4–7 months) and at 12 months (when followup stopped; range 10–14 months). We compared the z-scores by linear regression and calculated relative risks of being stunted or underweight. 

In the second approach, we studied the effect of VAS in the time windows where the BCG, DTP, and MV were the last vaccines to investigate whether the effect of neonatal VAS with BCG changed when the immune system was influenced sequentially by different vaccines (Figure 1). We focused on changes in weight for length which better catch short-term changes in growth [27]. The vaccine windows began when the child received the vaccine in question and ended one week after vaccination with a different vaccine (or at a chosen cut-off age if no subsequent vaccine was given). Growth in the BCG window was defined as the difference in the weight-for-length z-score from inclusion to within one week after the first DTP vaccine (DTP1); if no DTP was given, the last measurement before 4 months of age was used. Similarly, growth in the DTP window was defined as the difference in weight-for-length z-score between the first measurement after DTP1 and the last measurement within one week after the first MV. If the child had not received an MV before 12 months of age the last obtained measurement before/at 12 months of age was used. Finally, the growth in the MV window was defined as the difference in the first z-score measured after MV and the last measurement before/at 12 months of age (Figure 1). We also conducted the analysis on growth in the MV window stratified by reception of early MV at 4.5 months and 9 months of age or routine MV at 9 months. 

To contribute in one of the vaccines window analyses a child should thus have at least two measurements in the time window. The change in weight for length between the first and the last measurements in a vaccine window was compared by linear regression. A mixed-effect regression model for repeated measures was used to examine whether the effect of VAS differed between the vaccine windows. 

Others have found an effect of VAS on growth in vitamin-A-deficient children [28]. We did not measure vitaminAstatus at the time of supplementation. It has been shown consistently that vitamin A status varies with birth weight, that is, smaller newborns have poorer vitamin A status [29–32]. In addition boys have lower cord blood retinol concentration [29]. We therefore investigated whether socioeconomic status and weight in the first week of life modified the effect of VAS on weight and length for age at 6 and 12 months and on change in weight for length in the vaccine windows.

## 3. Results

We enrolled 808 children. Of these 490 were later enrolled in the early measles vaccine trial. At baseline the vitamin A and placebo groups were comparable (Table 1). Children included in the growth cohort, but never measured (*n* = 83), showed the same distribution of background factors (data not shown).

**Figure 2:**
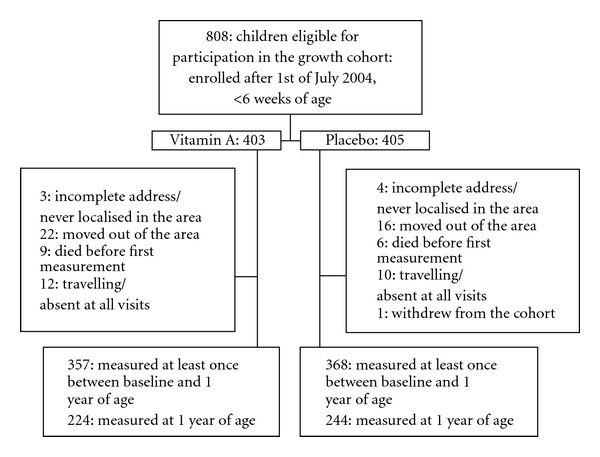
Flow of children through the study.

We aimed to visit the children approximately every 6 weeks. Mean time between two visits was 46.3 days (standard deviation (SD) = 9.3). The intervals between two visits did not differ by randomisation group (*P* = 0.46). An average of 68% of the children was found at home to be measured at these visits (VAS: 67%, Placebo: 68%). The majority of those not measured were travelling at the time of the visit. Of the 808 children included in the growth cohort, 725 (90%) were measured at least once between inclusion and 12 months of age. The remaining 83 were not measured for reasons stated in Figure 2. Twenty-seven children died before 1 year of age (19 VAS: 10 boys, 9 girls; 8 placebo: 5 boys, 3 girls), corresponding to a relative risk (RR) of loss to followup due to death for VAS versus placebo of 2.38 (1.06–5.39). 

### 3.1. First Approach: Anthropometric Status at 6 and 12 Months

At 6 months of age there was no overall effect of VAS at birth on length-for-age z-score (*P* = 0.58) or weight-for-age z-score (*P* = 0.79), and the effect of VAS did not differ between boys and girls, *P* = 0.28 for length for age and *P* = 0.25 for weight for age. VAS tended to increase the proportion of stunted girls, but to decrease the proportion of stunted boys (*P* for interaction between VAS and sex = 0.11) (Table 2). At 12 months there was no overall effect of VAS at birth on length for age (*P* = 0.28) or weight for age (*P* = 0.35) (Table 2). The majority of the children had attained a length below the reference median (90%), resulting in an average length for age of below −1 SD in both the VAS and the placebo groups. VAS tended to benefit the growth in boys during the first year of life but this was not found in girls (*P* for interaction between sex and VAS = 0.27 for being stunted and 0.06 for being underweight).

### 3.2. Second Approach: Growth by Vaccination Status

In the time window of the BCG vaccination there was an overall significant beneficial effect of VAS due to a significant beneficial effect for boys (Table 3). In the time window of the DTP vaccine there was no overall effect of VAS at birth, but a tendency for a negative effect was seen for girls (*P* = 0.12). In the time window of the MV there was no effect overall or in either sex. The overall effect of VAS in the BCG window was different from the effect in the DTP window (*P* = 0.03), but did not differ from the effect in the MV window (*P* = 0.11). Furthermore, the effect of neonatal VAS tended to differ between the DTP and MV windows for girls (*P* = 0.09), but not for boys (*P* = 0.67). The beneficial effect in the MV window may have been slightly stronger among the children who received an early MV and another one at 9 months of age. In none of the time windows the effect of neonatal VAS differed significantly between boys and girls (*P* for interaction between VAS and sex = 0.28, 0.18, and 0.61 in the BCG, DTP and MV windows, resp.).

Due to shortage of vaccine 21% of the children did not receive OPV at enrolment. The effect of neonatal VAS on growth at 6 or 12 months of age or in the vaccine windows did not differ by reception of OPV at enrolment (data not shown).

### 3.3. Growth by Markers of Vitamin A Status

Stratification by the background factors presented in Table 1 did not indicate that the effect of VAS on growth parameters at 6 or 12 months or in the vaccine windows differed by socioeconomic groups (data not shown). 

In tertiles of weight at enrolment for children enrolled in the first week of life, the effect of VAS was different in boys and girls. VAS tended to have a positive effect in the lightest girls in the BCG window immediately after supplementation, whereas the effect tended to be negative in the heavier girls (*P* = 0.10 for interaction between VAS and enrolment weight in the BCG window). In contrast, VAS tended to have a negative effect in the lightest boys and a positive in the heavier boys (*P* = 0.10 for interaction between enrolment weight and VAS in boys). This resulted in a statistically significant 3-way interaction between sex, VAS, and enrolment weight with respect to change in weight for length in the BCG window (*P* = 0.01). This effect was not sustained at 12 months of age (data not shown).

## 4. Discussion

Children in Guinea-Bissau do not fulfil their growth potential during the first year of life according to the WHO references. More than half were below minus 1 length-for-age z-score at 12 months of age. Overall there was no effect of neonatal VAS on growth at 6 and 12 months of age, but we observed a tendency towards a beneficial effect of VAS for boys but not for girls. The effect of VAS differed by vaccination status: VAS was beneficial as long as BCG was the most recent vaccine. The effect in the BGC window was significantly different from the effect in the DTP window where neonatal VAS tended to have a negative effect in girls. The effect of VAS tended also to differ between the DTP and MV windows for girls. 

### 4.1. Strengths and Limitations

We measured children enrolled in the randomised trial at home visits, limiting the dropout rate and minimising the risk of selection bias associated with children having to report to a health centre. However, the frequency of travelling and movements in urban Guinea-Bissau is high, and only around two-thirds of the children were measured during each round. For 10% we did not achieve a measurement at any time point during the first year of life. However, the baseline characteristics of these children did not differ from those of the measured children. In any growth study it could be argued that the results could be skewed if mortality during followup differed between the groups. In the present study mortality during followup was significantly higher in the vitamin A than in the placebo group, in particular among girls. This, if anything, would tend to mask a negative effect of VAS on growth in girls. 

The study of mortality revealed a strong interaction between season of supplementation and VAS, with VAS being detrimental for girls in the rainy season, and most beneficial for boys in the dry season [7]. Unfortunately, as this was an unexpected finding, it was not taken into account when planning the present study and all enrolments were in the rainy season Hence, we could study possible effect modification by season of enrolment.

### 4.2. Interpretation

We set out to examine the effect of VAS with BCG on the growth patterns in the first year of life including shifts in effect when the immune system is influenced sequentially by BCG, DTP, and then MV. As expected we found a positive effect in the BCG window. It seemed most pronounced for boys. In the time window of DTP, girls had a tendency for a negative effect of VAS, whereas boys still may have a slight benefit of being assigned to the VAS group. This may be due to a nonbeneficial interaction of vitamin A with DTP in girls, consistent with the mortality results [23]. The effect of VAS differed significantly between the BCG and the DTP windows, and there was a borderline significant difference between the DTP and MV windows in girls.

It could be argued that that positive effect of VAS in the BCG window was due to prevention/treatment of VAD, and this effect waned as vitamin A was consumed. However, this would not account for the tendency for a negative effect in the DTP window in girls. Others have found an effect of VAS on growth limited to groups with low vitamin A status [33], but this pattern is not supported by our data. In the present study boys had a more beneficial effect than girls in the BCG window; however, it was the boys with higher inclusion weights who seemed to benefit more from VAS in the BCG window. The opposite was the case for girls. If the effect is different in boys and girls, with a tendency for most beneficial effect in the least deficient boys and the most deficient girls immediately after supplementation, it seems unlikely that the beneficial effect of VAS at birth in certain subgroups was merely due to a reduction in VAD. Furthermore, if the effect of VAS on growth depended on the prevalence of VAD, we would expect a more pronounced difference between vitamin A and placebo among those of lower socioeconomic status [34–36]. We found no indication that a beneficial effect was seen in subgroups with worse socioeconomic status. Hence the effect of VAS on growth may be due to an interaction with the immune system, VAS affecting susceptibility to infections differently when BCG and DTP, respectively, have been administered as the most recent vaccine. 

## 5. Conclusion

The effect on growth of 50 000 IU vitamin A given at birth with BCG vaccine appears to be beneficial for boys, whereas it tended to be negative for girls who had received DTP. The mechanisms behind these effects are unknown, but are unlikely to be explained by prevention of vitamin A deficiency.

## Figures and Tables

**Table 1:** Baseline characteristics of all children included in the growth cohort by randomisation group.

	Vitamin A	Placebo
	Number (%)	Number (%)
Number	403	405
Sex (Male)	198 (49)	194 (48)
No oral polio vaccine at enrolment	88 (22)	83 (20)
Electricity in the household*		
Yes	142 (35)	140 (35)
No	246 (61)	261 (64)
Bathroom*		
Inside the house	63 (16)	56 (14)
Outside the house	324 (80)	343 (85)
None	0 (0)	2 (0)
Maternal education*		
Any	256 (64)	239 (59)
None	122 (30)	147 (36)
Birth order*		
Primipara	109 (27)	113 (28)
2nd-3rd	156 (39)	142 (35)
4th or higher	124 (31)	147 (36)

	Mean (SD)	Mean (SD)

Birth weight/kg	3.19 (0.44)	3.23 (0.40)
Weight at incl./kg	3.19 (0.51)	3.22 (0.51)
Weight for age at incl./z-score	−0.47 (0.95)	−0.38 (0.88)
Length at inclusion	50.2 (2.3)	50.1 (2.3)
Length for age at incl./z-score	−0.08 (1.00)	−0.12 (0.93)
Maternal age/years	25.0 (5.7)	25.1 (5.6)
Maternal arm circumference/mm	255 (33)	254 (30)

*Numbers do not add up due to a few with missing information.

**Table 2:** The effect of neonatal vitamin A supplementation (VAS) on weight and length parameters at 6 and 12 months of age overall and by sex.

	All				Boys				Girls			
	VAS	Placebo	Difference (95% CI)	RR (95% CI)	VAS	Placebo	Difference (95% CI)	RR (95% CI)	VAS. A	Placebo	Difference (95% CI)	RR (95% CI)
6 months of age												

*N*	308	308			149	150			159	158		
Age at measurement in days	181	182			181	182			181	182		
Length in cm	64.4	64.3	0.1 (−0.4; 0.49)		65.3	65	0.3 (−0.4; 0.9)		63.6	63.7	−0.1 (−0.7; 0.4)	
Length for age z-score (LAZ)	−0.97	−1.01	0.05 (−0.12; 0.22)		−1.04	−1.18	0.14 (−0.12; 0.41)		−0.90	−0.86	−0.04 (−0.25; 0.17)	
Stunted (LAZ <−2)	16%	16%		1.00 (0.69; 1.44)	19%	24%		0.81 (0.52; 1.25)	12%	8%	1.57 (0.79; 3.13)	
Weight in kg	7.59	7.61	−0.02 (−0.18; 0.14)		7.83	7.96	−0.13 (−0.35; 0.10)		7.36	7.28	0.08 (−0.13; 0.30)	
Weight for age z-score (WAZ)	−0.05	−0.03	−0.02 (−0.18; 0.14)		−0.12	0.00	−0.12 (−0.36; 0.12)		0.02	−0.06	0.08 (−0.16; 0.31)	
Underweight (WAZ <−2)	3%	2%		1.66 (0.61; 4.53)	3%	2%		1.68 (0.42; 0.6.90)	3%	2%		1.66 (0.40; 6.81)

12 months of age												

*N*	224	244			102	121			122	123		
Age at measurement in days	362	363			359	363			365	363		
Length in cm	71.7	71.6	0.1 (−0.3; 0.6)		72.4	72.1	0.2 (−0.4; 0.9)		71.1	71.0	0.1 (−0.5; 0.7)	
Length for age z-score (LAZ)	−1.20	−1.30	0.10 (−0.08; 0.28)		−1.32	−1.48	0.16 (−0.12; 0.43)		−1.11	−1.13	0.02 (−0.21; 0.25)	
Stunted (LAZ <−2)	21%	24%		0.85 (0.60; 1.19)	25%	34%		0.75 (0.49; 1.14)	16%	15%		1.12 (0.63; 2.01)
Weight in kg	8.86	8.79	0.08 (−0.14; 0.29)		9.11	9.01	0.10 (−0.20; 0.39)		8.66	8.57	0.09 (−0.22; 0.41)	
Weight for age z-score (WAZ)	−0.44	−0.54	0.10 (−0.11; 0.31)		−0.54	−0.66	0.12 (−0.17, 0.42)		−0.37	−0.43	0.06 (−0.23, 0.35)	
Underweight (WAZ <−2)	7%	9%		0.74 (0.39, 1.38)	5%	13%		0.39 (0.14, 1.04)	8%	6%		1.44 (0.57, 3.66)

**Table 3:** The effect of neonatal vitamin A supplementation (VAS) on changes in weight for length by vaccination status overall and by sex.

Vaccine windows	All			Boys			Girls		
	VAS	Placebo	Difference (95% CI)	VAS	Placebo	Difference (95% CI)	VAS	Placebo	Difference (95% CI)
BCG window									

*N*	174	176		86	76		88	100	
Change in z-score for weight for length	1.47	1.17	**0.31 (0.03; 0.59)**	1.71	1.26	**0.45 (0.05; 0.86)**	1.24	1.09	0.14 (−0.25; 0.53)

DTP window									

*N*	292	308		140	150		152	158	
Change in z-score for weight for length	−0.24	−0.16	−0.08 (−0.28; 0.12)	−0.24	−0.30	0.06 (−0.24; 0.35)	−0.24	−0.03	−0.21 (−0.48; 0.06)

MV window									

*N*	189	180		91	83		98	97	
Change in z-score for weight for length	−0.33	−0.37	0.04 (−0.12; 0.20)	−0.32	−0.31	0.01 (−0.24; 0.23)	−0.34	−0.42	0.08 (−0.15; 0.31)

MV window stratified: MV at 4.5 + 9 months									

*N*	81	66		37	30		44	36	
Change in z-score for weight for length	−0.50	−0.62	0.12 (−0.14; 0.38)	−0.49	−0.60	0.11 (−0.30; 0.52)	−0.50	−0.63	0.13 (−0.21; 0.47)

MV window stratified: MV at 9 months									

*N*	108	114		54	53		54	61	
Change in z-score for weight for length	−0.21	−0.23	0.02 (−0.19; 0.23)	−0.20	−0.15	−0.05 (−0.32; 0.22)	−0.22	−0.30	0.08 (−0.23; 0.40)
